# Mango “Ataulfo” Peel Extract Improves Metabolic Dysregulation in Prediabetic Wistar Rats

**DOI:** 10.3390/life12040532

**Published:** 2022-04-05

**Authors:** Alejandra M. Preciado-Saldaña, Jesús Abraham Domínguez-Avila, Jesús Fernando Ayala-Zavala, Humberto F. Astiazaran-Garcia, Marcelino Montiel-Herrera, Mónica A. Villegas-Ochoa, Gustavo A. González-Aguilar, Abraham Wall-Medrano

**Affiliations:** 1 Coordinación de Tecnología de Alimentos de Origen Vegetal, Centro de Investigación en Alimentación y Desarrollo (CIAD), A. C. Carretera Gustavo Enrique Astiazarán Rosas No. 46, La Victoria, Hermosillo 83304, Sonora, Mexico; alejandra.preciado@estudiantes.ciad.mx (A.M.P.-S.); abrahamdominguez9@gmail.com (J.A.D.-A.); jayala@ciad.mx (J.F.A.-Z.); mvillegas@ciad.mx (M.A.V.-O.); 2 Cátedras-CONACYT, Centro de Investigación en Alimentación y Desarrollo (CIAD), A. C. Carretera Gustavo Enrique Astiazarán Rosas No. 46, La Victoria, Hermosillo 83304, Sonora, Mexico; 3 Coordinación de Nutrición, Centro de Investigación en Alimentación y Desarrollo (CIAD), A. C. Carretera Gustavo Enrique Astiazarán Rosas No. 46, La Victoria, Hermosillo 83304, Sonora, Mexico; hastiazaran@ciad.mx; 4 Departamento de Medicina y Ciencias de la Salud, Universidad de Sonora, Hermosillo 83000, Sonora, Mexico; marcelino.montiel@unison.mx; 5 Instituto de Ciencias Biomédicas, Universidad Autónoma de Ciudad Juárez, Anillo Envolvente del Pronaf y Estocolmo S/N, Ciudad Juárez 32315, Chihuahua, Mexico

**Keywords:** prediabetes, phenolic compounds, mango peel, byproducts, anti-diabetic effects

## Abstract

The hypoglycemic effect of functional phytochemicals has been evaluated in diabetic rodents but scarcely in its premorbid condition (prediabetes; PD). This study aimed to evaluate a mango (cv. Ataulfo) peel hydroethanolic (20:80) extract (MPE) for in vivo glycemic/lipidemic-normalizing effect and in vitro enzyme inhibitory (α-amylase/α-glucosidase) activity. The polyphenolic MPE (138 mg EAG.g^−1^, mainly gallic acid and mangiferin) with antioxidant capacity (DPPH• 34 mgTE.g^−1^) was fed to PD rats (induction: high-fat diet (60% energy) + single dose streptozotocin (35 mg·kg^−1^), 4 weeks). At the 8th week, fasting glycemia (FG), oral glucose tolerance test, and insulin sensitivity indexes (HOMA-IR, HOMA-β) > blood lipid-normalizing effect were documented as healthy controls > MPE > disease (PD) controls, which was possibly related to the extract’s concentration–response in vitro enzyme inhibitory activity (IC_50_ ≈ 0.085 mg·mL^−1^). MPE is a rich source of glucose-lowering phytochemicals for the primary prevention of type 2 diabetes.

## 1. Introduction

Adult prediabetes (PD) is clinically defined when fasting hyperglycemia (FG, 101–125 mg·dL^−1^), abnormal glycated hemoglobin (HbA1c, 5.7% and 6.4%), and 75 g oral impaired glucose tolerance (140–199 mg·dL^−1^ 2 h after) concur [1,2,3], but the values are not high enough for a type 2 diabetes (T2D) formal diagnosis. Due to its silent subclinical course, PD remains untreated before it develops into T2D, placing the patient at an increased risk for other chronic illnesses, including micro/macrovascular complications [2]. It is estimated that nearly 10% of people with PD may develop T2D within a year, while an additional 70% will eventually suffer from it within the next 5 to 10 years [4]. Fortunately, lifestyle modifications (i.e., healthy eating, regular physical activity) help to restore normal glycemia in people with PD or T2D [5]. In particular, the American Diabetes Association (ADA) recommends consuming fruits and vegetables, due to their functional phytochemical content (i.e., phenolic compounds (PC), carotenoids, alkaloids, peptidoglycans, phytosterols, etc.) with glucose-lowering effect [1,6]. 

Most phytochemicals not only exert antioxidant activity but also other anti-diabetic bioactivities, including enzyme inhibition, insulin secretagogue, molecular signaling, and epigenetic action, which may modify the PD-to-T2D *dysregulated glycemia* continuum and other hypermetabolic features, including hyperlipidemia [7,8,9]. Particularly, PC regulate carbohydrate digestion and absorption, satiety induction, insulin secretion, and molecular signaling, all of them contributing to glycemic/insulinemic homeostasis [10,11,12]. Studies unveiling the molecular hypoglycemic mechanisms of PC are of great interest in diabetology, but those focused on their effect on PD are quite recent [13,14]. The continuous research and development of safer pharmacological and complementary (e.g., nutraceutical) treatments to stop the progression of PD-to-T2D can significantly reduce the incidence of the latter.

Mango (*Mangifera indica* L.) is one of the most consumed tropical fruits worldwide. Its industrial processing generates large amounts of by-products, such as its peel, most of which is directly discarded. The reintroduction of agro-industrial by-products into the food processing chain has been recently considered for various purposes by using them as unconventional sources of functional phytochemicals. Mango cv. ‘Ataulfo’ peel is rich in mangiferin (xanthonoid), gallic acid, gallotannins, quercetin, and catechin, with proven health effects, including anti-diabetic actions [15,16]. Recent studies have shown that mango phenolics obtained from different tissues (including its peel) can exert significant improvements on impaired glycemia and insulin resistance through different mechanisms [17,18], although their effects in PD rodents have not been studied. Thus, the objective of this work was to characterize a phenolic-rich ethanolic extract from mango peel and evaluate its effects on various biochemical parameters in a murine model of a high-fat diet, low-dose streptozotocin-induced PD.

## 2. Materials and Methods

### 2.1. Materials and Reagents

Mango cv. ‘Ataulfo’ fruits were purchased in a local market (Hermosillo, Son., Mexico; 29°6′9.4″ N 110°58.639′ W) and transported to the laboratory. Ripened fruits (stage 4) were selected as described previously [19]. Sanitized fruits were carefully peeled with a sharp knife, and the peel, pulp, and seed were separated. The peel was frozen (−35 °C), freeze-dried (−50 °C) in a freeze-drier (Labconco, Kansas City, MO, USA), and stored in amber bags at −80 °C until later use. Solvents used for the extraction of phenolic compounds and mobile phases (ethanol, methanol, and formic acid) were obtained from JT-Baker (Mexico City, Mexico). Pure standards of phenolic compounds, streptozotocin, α-amylase (E.C. 3.2.1.1), and α-glucosidase (E.C. 3.2.1.20) were obtained from Sigma-Aldrich (St. Louis, MO, USA).

### 2.2. Mango Peel Extract (MPE)

#### 2.2.1. Extract Preparation

Bioactive compounds were extracted from freeze-dried mango peel, using a solution of ethanol/water (80:20 *v*/*v*). Ethanol solution was added to mango peel (1:10 *w*/*v*), sonicated for 30 min (Bransonic, Danbury, CT, USA), and centrifuged at 14,000 rpm for 15 min at 4 °C (Allegra 64R Centrifuge, Beckman Coulter, Indianapolis, IN, USA). Then, the supernatant was filtered, and the procedure was repeated twice. Ethanol was removed by rotary evaporation and the remaining water by freeze drying. The dry mango peel extract (MPE) obtained was stored in amber vials at −80 °C and used for all subsequent in vitro and in vivo experiments.

#### 2.2.2. Total Phenolic Content

The Folin–Ciocalteu colorimetric assay was used to determine the total phenolic content of MPE [20]. Results were expressed as mg of gallic acid equivalents (GAE).g^−1^.

#### 2.2.3. Total Flavonoids

The total flavonoid content of MPE was quantified using a colorimetric assay, as described by Quirós-Sauceda et al. [20]. Briefly, flavonoids were extracted with 5% NaNO_2_, 10% AlCl_3_, and l M NaOH, and measured spectrophotometrically at 510 nm using quercetin as standard. Results were expressed as mg of quercetin equivalents (QE).g^−1^.

#### 2.2.4. Antioxidant Capacity

The antioxidant capacity of MPE was measured using three different methods: the 2,2-diphenyl-1-picrylhydrazyl (DPPH•) assay, the ferric-reducing antioxidant power assay (FRAP), and the oxygen radical absorbance capacity (ORAC) assay. Trolox (6-hydroxy-2,5,7,8-tetramethylchroman-2-carboxylic acid) was used as standard, and results are expressed as mg Trolox equivalents (TE).g^−1^. The DPPH• assay was performed as reported by Quirós-Sauceda et al. [20], FRAP and ORAC were performed as described by Quirós-Sauceda et al. [21].

#### 2.2.5. Chromatographic Identification of Phenolic Compounds

The extracted phenolic compounds were identified and quantified in a diode array detector ultra-resolution liquid chromatography system (UPLC-DAD; ACQUITY, Waters Corp., Milford, MA, USA), as previously reported by Velderrain-Rodríguez et al. [22]. Separation was performed on a BEH C_18_ column (3.0 mm × 100 mm, 5 µm, Waters) at 60 °C. The mobile phases were 0.5% formic acid and methanol. The eluted compounds were identified by comparing their retention times and absorption spectra with their respective commercial standards and were quantified using standard curves prepared with the same standards.

#### 2.2.6. Reducing Sugars

Reducing sugars were quantified according to the methodology reported by Contreras-Jácquez et al. [23]. Results are expressed as mg glucose equivalents (GE).g^−1^.

### 2.3. Enzyme Inhibition 

#### 2.3.1. α-Amylase Activity Assay

The α-amylase activity assay was performed according to Ren et al. [24], using corn starch as substrate (1 g.100 mL^−1^, prepared in 0.2 M, pH 6.8 PBS). Serial dilutions (0.02, 0.04, 0.06, 0.08, and 0.1 mg·mL^−1^) of MPE were prepared in phosphate buffer/dimethyl sulfoxide (DMSO, 9:1). Then, 250 µL of each MPE solution was mixed with 250 µL of α-amylase (300 U.mL^−1^ in PBS) in a glass tube. Acarbose was used as positive control for this assay at the same concentrations as the MPE. All mixtures were then placed in a 37 °C water bath for 10 min at 150 rpm, and 500 µL of starch solution was added. After the incubation period, 1 mL of a 1% dinitrosalicylic acid (DNS) solution was added, the tubes were heated in a water bath (100 °C) for 5 min, cooled to room temperature and then, 25 mL of deionized water were added. Their absorbance was measured at 540 nm, and the inhibitory effects of MPE on α-amylase were calculated and expressed as half-maximal inhibitory concentration (IC_50_, mg·mL^−1^).

#### 2.3.2. α-Glucosidase Activity Assay

The α-glucosidase activity assay was performed according to Ren et al. [24] with some modifications. An α-glucosidase solution (60 U/mL), *p*-nitrophenol-α-d-glucopyranoside (*p*NPG, 5 mM), and different concentrations of MPE (0.02, 0.04, 0.06, 0.08, and 0.1 mg·mL^−1^) were prepared in phosphate buffer (0.2 M, pH 6.8). Acarbose was used as positive control in this assay. Afterwards, 100 µL of different mango peel extract solutions and 50 µL of enzyme solution were added into a microplate well, mixed, and incubated for 10 min at 37 °C. After the incubation period, 50 µL of *p*NPG solution was added, and the reaction was carried out for 30 min at 37 °C. Immediately after, 100 µL of 0.2 M sodium carbonate (Na_2_CO_3_) was added, incubated for 5 min under gentle mixing, and the absorbance was measured at 405 nm. The absorbances were used to calculate the inhibitory effects of MPE on α-glucosidase, and expressed as IC_50_.

### 2.4. Bioassay 

#### 2.4.1. Animals and Diets

All experiments involving animals were reviewed and approved by the Bioethics Research Committee of the Research Center for Food and Development, where they were performed (CEI/011-2/2020). They were carried out according to the National Research Council’s Guide for the Care and Use of Laboratory Animals and the Mexican NOM-062-ZOO-1999. Twenty-six male Wistar rats weighing 200 ± 20 g were obtained from the Department of Medicine and Health Sciences of the University of Sonora (Mexico). The animals were individually housed in metal cages with 12 h light–dark periods and ad libitum access to food and water. Three diets were made: a standard chow diet, whose composition mimicked the 5001-maintenance diet (3.5 kcal.g^−1^), the same standard diet supplemented with MPE (5 g.kg^−1^), and a non MPE-supplemented high-fat diet (HFD, 64% kcal from fat).

**Figure 1:**
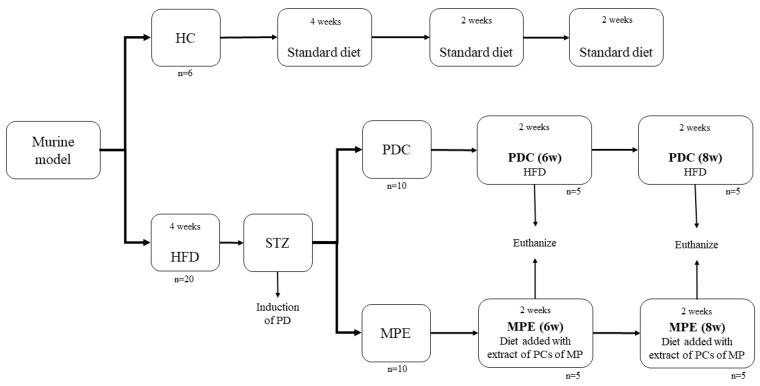
Overview of the experimental protocol followed in the in vivo part of the study. HC: healthy control; PDC: prediabetes control; MPE: mango peel ethanolic extract group; HFD: high-fat diet; STZ: streptozotocin.

Rats were divided into three groups: a healthy control group (HC, n = 6) fed with the standard chow diet for 28 d, a disease (PD) control group (PDC; n = 10) fed with HFD for 56 d, and the experimental group fed with the HFD for 28 days and then with the standard chow diet supplemented with MPE (MPE; n = 10) for another 28 days (Figure 1).

#### 2.4.2. PD Induction

Rats consumed the HFD for 28 d and received a single dose of streptozotocin (STZ, 35 mg·kg^−1^) on day 24, which leads to the development of PD in male Wistar rats (unpublished data). The animals were weighed once weekly throughout the experiment, while food consumption was measured daily.

#### 2.4.3. Biochemical Parameters

FG at 28 d (basal) and 56 d (end) was measured after an overnight fast and under anesthesia (sodium pentobarbital 120 mg·kg^−1^ BW, Pisabental^®^ PISA Laboratories, Mexico City, Mexico), by taking a capillary blood sample from the tail, before the animals were euthanized. A commercial glucose meter was used for this purpose (One Touch Ultra mini, LifeScan, Milpitas, CA, USA). After confirming the complete absence of reflexes, blood samples were also collected by cardiac puncture into EDTA-containing tubes (Vacutainer, Becton-Dickinson, Plymouth, England), until exsanguination. Blood samples were centrifuged (4000 rpm, 25 °C, 15 min), and the recovered plasma was stored at −80 °C for subsequent analyses.

Insulinemia was quantified with a rat-specific ELISA kit (Sigma-Aldrich). Total cholesterol (TC), HDL cholesterol (HDL-c), and triacylglycerides (TAG) were quantified using commercially available colorimetric kits (Stanbio, Boerne, TX, USA). LDL cholesterol (LDL-c) and VLDL cholesterol (VLDL-c) were calculated according to Macho-González, et al. [25] (Equations (1) and (2)).

LDL-c = TC-(TAG/5)-HDLc(1)

VLDL-c = TAG/5(2)

#### 2.4.4. Oral Glucose Tolerance Test (OGTT)

An oral glucose tolerance test (OGTT) was performed on the last day of the experiment (before euthanasia) as described by Gondi and Prasada-Rao [26]. The animals were fasted overnight, fasting glycemia was quantified (as previously mentioned), and a glucose solution was orally administered to achieve a load of 2 g/kg body weight. Glycemia was quantified from the tail vein at 0, 15, 30, 60, 60, 90, 120, and 150 min using a commercial glucose meter. Total glycemic responses to the OGTT were calculated from the areas under the curve (AUC).

#### 2.4.5. Insulin Sensitivity 

Three indexes were used for the evaluation of insulin sensitivity, which were calculated from the animals’ glycemia and insulinemia. Homeostatic model assessment of insulin resistance (HOMA-IR), pancreatic β-cell reserve (HOMA-B), and quantitative insulin sensitivity index (QUICKI) were calculated according to Equations (3)–(5), as reported by Wang et al. [27], Nurdiana et al. [28], and Barman and Srinivasan [29].

HOMA-IR = [(Fasting insulin ((µIU)/mL) × fasting glucose (mmol/L))/22.5](3)

HOMA-B = (20 × fasting insulin ((µIU)/mL))/(fasting glucose (mmol/L) − 3.5)(4)

QUICKI = [1/(log fasting insulin ((µIU)/mL) + log fasting glucose (mmol/L)(5)

#### 2.4.6. Statistical Analysis

Results were analyzed by a one-way analysis of variance (ANOVA) and Tukey–Kramer’s test to identify differences between treatments (*p* < 0.05), using the NCSS 2007 software (Kaysville, UT, USA).

## 3. Results and Discussion

### 3.1. Chemical Profile of MPE

Before the in vivo effects of the MPE were determined, it was first necessary to thoroughly characterize its bioactive composition, antioxidant activity, and effects on digestive enzymes. Since the extract was intended for animal consumption, ethanol was used as extraction solvent, due to its safety, as compared to methanol or others [30].

As expected, the characterization of the MPE showed evidence of a high concentration of bioactive compounds. The main phytochemicals present were phenolic compounds (137.68 ± 0.55 mg GAE/g) and total flavonoids (18.55 ± 0.19 mg QE/g). Due to its phenolic-rich nature, it also had high antioxidant capacity (mg TE.g^−1^: DPPH• (33.54 ± 0.38), FRAP (37.13 ± 1.95) and ORAC (23.71 ± 0.73)) as compared to other tropical fruit pulps [20] and mango cv. ‘Badami’ peel extract [26]. MPE also had 374.95 mg GE/g of reducing sugars, which is a significantly lower value (approximately three times less) than that found in its pulp [21]. Ruiz-Montañez et al. [31] have shown that ethanol/water extraction is more effective for the extraction of phenolic compounds as compared to other solvents. Specifically, ethanol is more efficient when extracting mangiferin from mango peel, further supporting its use in the present work, since this compound is considered highly bioactive in biological models.

The identification (Figure S1) and quantification (Table S1) of phenolic compounds by UPLC-DAD revealed the presence of seven individual molecules: namely, gallic acid, catechin, chlorogenic acid, epicatechin, quercetin derivatives, and mangiferin, the latter being the main phenolic component (Table S1, Figure S1). This coincides with works of other authors, where exhaustive identifications have previously been carried out in mango peel. For example, Pacheco-Ordaz, et al. [15] reported that after a methanolic extraction, the free phenolic fraction mainly contained gallotannins, mangiferin, and flavonoids.

### 3.2. Enzyme Inhibitory Activity of MPE

The phenolic compounds found in the extract used in the present work have shown different beneficial effects in some in vitro and in vivo models. For example, mangiferin has been shown to be a potential inhibitor of digestive enzymes such as α-amylase and α-glucosidase [32]. It has also been proven that this compound can promote glucose utilization and metabolism in a dose-dependent manner [33].

The use of α-amylase and α-glucosidase inhibitors is one common strategy whose effectiveness has been established. Their mechanism of action is based on delaying and/or reducing intestinal glucose absorption, thereby minimizing glycemic and insulinemic spikes. The use of inhibitors derived from natural sources has been of particular interest in modern times, which can be used as an alternative to synthetic molecules or to complement their effects. This is due to some side effects exerted by the synthetic options, which can be potentially avoided with compounds already found in everyday diet. Phenolic compounds have been reported to be effective suppressors of postprandial hyperglycemia at different concentrations [34].

The effect of MPE on starch digestion was evaluated, according to its ability to inhibit the activities of α-amylase and α-glucosidase.

**Figure 2:**
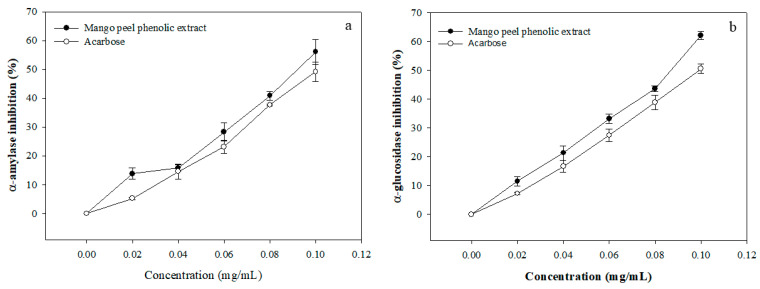
Activity of (**a**) α-amylase and (**b**) α-glucosidase in the presence of various concentrations of mango peel ethanolic extract (MPE) and acarbose.

As compared with acarbose, one of the main enzyme inhibitors, MPE showed comparable effectiveness. Its inhibitory effect increased concentration-dependently (Figure 2) with an IC_50_ of 0.089 mg·mL^−1^ and 0.080 mg·mL^−1^ for α-amylase and α-glucosidase, respectively, suggesting a similar effect on both enzymes.

These IC_50_ values are higher than those found by other authors in a mango cv. ‘Badami’ peel extract [26], where values of 4.0 and 3.5 μg.mL^−1^ were reported. In contrast, Irondi et al. [35] prepared a mango seed phenolic extract, and they report IC_50_ values of 0.74 and 0.34 mg·mL^−1^ for α-amylase and α-glucosidase, respectively, which could indicate that although mango seed and peel have a similar phenolic profile, those in the peel have a greater potential to inhibit the activity of carbohydrate-digesting enzymes.

Several studies have been carried out in which different phenolic-rich extracts from various plant sources have been used as potential inhibitors of α-amylase and α-glucosidase. Li et al. [36] evaluated a persimmon phenolic-rich extract on starch digestion in vitro and *in vivo*, and they report that it was able to inhibit α-amylase at a concentration of 0.35 mg·mL^−1^.

The in vitro effects of the bioactive compounds present in the extract analyzed in this study may be attributed to specific molecules. Mangiferin is one of the most likely ones, since previous studies have reported its effects as a potential α-amylase inhibitor [37], while it was also the most abundant molecule found herein. However, it is also possible that the inhibitory effects may also be due to the presence of other minor compounds found in the extract as well as interactions between them. The bioactivities of mangiferin, and the extract in general, are also likely to extend beyond in vitro models, according to its insulin-stimulating effects that can improve glycemic control [38].

### 3.3. Effects of MPE in PD Rats 

The HC group had a higher diet consumption during the eight weeks of experimental period as compared to the HFD-fed groups (Table 1, Figure S2). Diet consumed by HFD-fed rats was similar during the first five experimental weeks, although the PDC group had a decrease from the sixth to the eight weeks. The animals’ weight gain throughout the experimental period was similar, except for the PDC group, which had a lower weight gain during the first five weeks, although the weight was similar in all groups by the end of the experiment. After PD was induced on the PDC and MPE groups (four weeks of consuming an HFD and low-dose streptozotocin injection), fasting glycemia was measured; the results are shown in Table 1. The blood glucose levels of all animals were either in the normal (≤100 mg·dL^−1^) or prediabetic range (101–125 mg·dL^−1^). 

Most groups had similar glycemia values, except for the MPE (6 w) group, which was significantly higher than the HC, albeit they were both within the normal range. After two or four more weeks of experimental treatment after PD was induced (groups 6 w and 8 w), fasting glycemia was again measured. The values obtained were numerically similar to those of the previous measurement (and within the same normal or prediabetic ranges), but the PDC (8 w) and MPE (6 w) groups now had significantly higher values than the HC and PDC (6 w) groups.

Glycemia by itself may not be sufficient to conclusively establish PD; thus, insulinemia was measured at the same time as glycemia of the previous period in order to determine insulin sensitivity indexes. Insulinemia had increased two weeks after the low-dose streptozotocin dose (PDC (6 w) group) but not enough to be significantly different than the HC group. Four weeks after the streptozotocin dose (PDC (8 w) group), the highest insulinemia was recorded, which was significantly different than the mango-treated groups (MPE (6 w) and (8 w)). The insulinemia of the MPE groups was numerically and statistically similar to that of the HC group, suggesting a targeted effect exerted by the treatment. Therefore, it is apparent that although glycemia was within the normal or prediabetic range, insulinemia was significantly affected by the combined effect of the HFD and streptozotocin injection, which the mango-derived bioactives were able to restore to normal values.

Regarding the insulin sensitivity indexes (Table 1), the behavior of HOMA-IR, the parameter used to determine insulin resistance, was similar to that of insulinemia, further confirming the normalizing effects of the mango-based treatment. In contrast, the effects of PD induction and experimental treatments were not as evident on HOMA-B and QUICKI. Insulin concentration plays the most important role in maintaining glycemic homeostasis; thus, our results suggest that that the bioactive compounds present in mango peel were able to attenuate the compensatory hyperinsulinemia that occurs during PD even while glycemia was within normal or near-normal values. Various studies have suggested that mangiferin is the main mango peel-derived compound with such a significant anti-diabetic effect, according to its ability to promote insulin sensitivity, in addition to effects on the activity of digestive enzymes, which can indirectly alter glycemia and, therefore, insulin sensitivity [39].

PD is a reversible condition; natural alternatives are currently being sought that, together with lifestyle changes, can help prevent its progression. Other compounds have also been shown to exert similar effects: for example, flavonoids contained in the extract, such as quercetin, have been shown to have anti-diabetic effects, according to a mechanism of action related to promoting insulin secretion, regeneration of pancreatic islets, and increased peripheral sensitivity to the hormone [40]. Others have also shown that quercetin-treated (intraperitoneally) murine models of obesity had improved insulin sensitivity by a mechanism related to the flavonoid binding to GLUT4 and promoting cellular glucose uptake through this transporter [41]. Quercetin can also aid in controlling fasting and postprandial glycemia and insulinemia as well as the prevention of compensatory hyperplasia and preservation of pancreatic β-cell mass in in vivo models [32,33]. Gallic acid is another relevant molecule due to its antioxidant properties that could potentially prevent chronic diseases [18,42]. Latha and Daisy [43] also reported that it may be a potential insulin secretagogue, according to its ability to improve cell regeneration and insulin secretion in a murine model of diabetes. Thus, the bioactive composition of the extract, and in particular its phenolic compounds, appear to be ideal to improve on some markers of PD.

Comparable results to the ones reported herein have been found by other authors when using a mango peel extract (200 mg·kg^−1^ body weight) to improve glycemia in streptozotocin-induced diabetic rats [26]. These effects may be mainly attributed to its high mangiferin and flavonoid content (Table S1). Cázares-Camacho et al. [44] used a diet supplemented with mango peel and pulp as pretreatment before diabetes induction in a murine model, and they showed better glycemic improvements than rats that consumed it once diabetes had been induced. Additionally, when using a hydroalcoholic extract of phenolic compounds from mango leaves (1 g.kg^−1^ body weight/d), a significantly decreased glycemia was reported after two weeks of treatment [45]. On the other hand, the use of phenolic-rich extracts from other plant sources has also been reported; for example, an extract of *European Caralluma* (500 mg·kg^−1^ of body weight) decreased the glycemia of diabetic rats [46].

**Figure 3:**
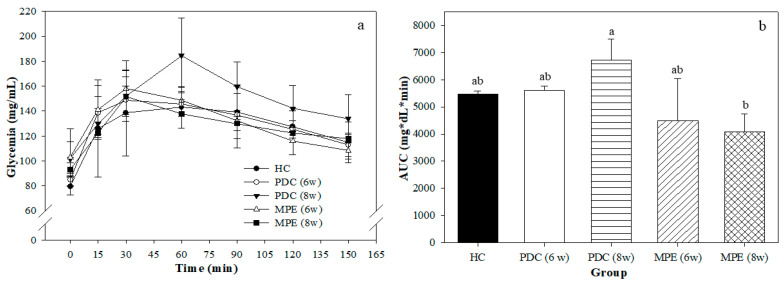
(**a**) Oral glucose tolerance test (OGTT) and (**b**) their corresponding areas under the curve (AUC). Different superscript letters indicate significant differences (*p* < 0.05). HC: healthy control; PDC (6 w) and (8 w): prediabetes control group after 6 and 8 weeks of experimental period, respectively; MPE (6 w) and (8 w): mango peel ethanolic extract (MPE) group after 6 and 8 weeks of experimental period, respectively.

Figure 3a shows the OGTT that was performed on the final day of the experimental period. The maximum glucose peak was found at 30 min in most groups, except for the PDC (8 w) group, which was delayed until 60 min. 

This suggests that this group had an altered glucose metabolism, according to its inability to exert a timely response to an oral glucose load. This finding is further corroborated with the AUC (Figure 3b), which shows a significant tendency toward an increase (although there were no significant differences with the HC) and a significantly higher value than that of the MPE (8 w) group. Glucose uptake capacity was improved in the MPE group as compared to the PDC group, which suggests that the extract of phenolic compounds from mango peel shows a postprandial hypoglycemic effect. Various anti-diabetic effects of phenolic compounds have been suggested, which may be exerted due to a combination of changes on glucose absorption, or indirect effects derived from microbiota modulation or others [47,48]. Some in vivo studies performed in diabetic animals have shown that the plant-derived derived polyphenols improve glucose absorption and glycemic response [48].

Finally, the lipid profile of the PDC (6 w) and PDC (8 w) groups were significantly altered as compared to the HC group (Table 1), while the MPE treatment was able to normalize, most notably, total cholesterol, VLDL, and triacylglycerides. Similarly, to hyperglycemia, hyperlipidemia affects the function of pancreatic β-cells, thereby contributing to impaired glucose homeostasis, and it is a significant risk to the development of PD and, consequently, type 2 diabetes. Safdar et al. [49] evaluated different doses of mango peel extract and concluded that a medium or high dose of the treatment (150 or 300 mg·kg^−1^ of body weight) can impart cardioprotective actions in rats due to improvements in the animals’ lipid profile in addition to the anti-diabetic effects. Thus, normalizing glycemia, insulinemia, and the lipid profile may be beneficial health effects, which can altogether mitigate the development of PD, T2D, and other chronic diseases.

## 4. Conclusions

In this study, a mango peel ethanolic extract (MPE) rich in a wide range of phytochemicals with a presumably anti-diabetic effect was prepared and evaluated in vitro (α-amylase and α-glucosidase inhibition assay) and in vivo (prediabetic (PD) rat model). Results indicate that the blood glucose > lipid normalizing effect of MPE, as compared to healthy and non-PD supplemented rats, could be partially related to MPE’s enzyme inhibitory activity (IC_50_ ≈ 0.085 mg·mL^−1^). Moreover, our data unveiled the ability of MPE as a preventive anti-diabetic acid when consumed in its premorbid phase (PD). However, to support stronger evidence-based conclusions on the benefits of MPE in a PD state, a third control PD group fed a normal (chow) diet not supplemented with MPE would have been necessary. Preliminary observations on such an experimental group have demonstrated that the transient PD state seems to be strongly associated with reduced food palatability and overall intake, which deserves a deeper physiological exploration in the near future.

## Figures and Tables

**Table 1:** Biological response of experimental groups.

Parameter	HC	PDC (6 w)	PDC (8 w)	MPE (6 w)	MPE (8 w)
Food intake (g·d^−1^)	23.2 ± 0.7 ^a^	14.6 ± 2.2 ^b^	14.6 ± 1.4 ^b^	17.8 ± 3.0 ^b^	17.5 ± 2.6 ^b^
Body weight (g)	344.6 ± 20.2 ^a^	267.4 ± 33.2 ^b^	308.0 ± 64.3 ^a^	326.4 ± 10.7 ^a^	299.0 ± 18.8 ^a^
FG (mg·dL^−1^)_Basal_	82.0 ± 2.6 ^b^	91.8 ± 9.0 ^ab^	111.8 ± 12.3 ^a^	90.0 ± 9.2 ^ab^	84.8 ± 2.2 ^b^
FG (mg·dL^−1^)_End_	83.8 ± 3.1 ^b^	84.6 ± 4.6 ^b^	114.4 ± 13.5 ^a^	89.2 ± 9.6 ^ab^	94.0 ± 5.2 ^ab^
FI (µUI·mL^−1^)_End_	14.2 ± 2.6 ^b^	18.5 ± 2.6 ^ab^	19.1 ± 6.1 ^a^	14.5 ± 3.0 ^b^	15.8 ± 2.0 ^b^
HOMA-IR	3.2 ± 0.7 ^b^	4.2 ± 0.5 ^ab^	5.8 ± 2.0 ^a^	3.4 ± 0.6 ^b^	4.0 ± 0.3 ^b^
HOMA-β	185.2 ± 15.8 ^b^	242.9 ± 64.8 ^a^	118.0 ± 43.3 ^c^	174.2 ± 71.2 ^bc^	152.6 ± 43.1 ^bc^
QUICKI	0.5 ± 0.0	0.5 ± 0.0	0.5 ± 0.0	0.5 ± 0.0	0.5 ± 0.0
TC (mg·dL^−1^)	64.0 ± 13.1 ^c^	81.9 ± 11.1 ^b^	96.7 ± 10.8 ^a^	67.0 ± 4.0 ^c^	76.6 ± 5.0 ^b^
HDL-c (mg·dL^−1^)	11.8 ± 1.1 ^a^	7.6 ± 0.7 ^bc^	5.5 ± 1.2 ^c^	8.5 ± 0.6 ^b^	8.0 ± 0.3 ^b^
LDL-c (mg·dL^−1^)	21.5 ± 13.0 ^c^	46.9 ± 7.5 ^b^	63.1 ± 8.9 ^a^	42.1 ± 7.1 ^bc^	50.5 ± 4.4 ^ab^
VLDL-c (mg·dL^−1^)	17.1 ± 4.1 ^b^	27.5 ± 5.6 ^a^	28.2 ± 3.6 ^a^	16.4 ± 5.1 ^b^	18.1 ± 1.0 ^b^
TAG (mg·dL^−1^)	86.7 ± 12.0 ^b^	137.2 ± 22.7 ^a^	140.8 ± 14.3 ^a^	81.8 ± 6.7 ^b^	90.6 ± 5.5 ^b^

Results are expressed as mean ± standard deviation (n = 6 rats.group^−1^). Different superscript letters in a same row indicate statistical differences (*p* < 0.05). Prediabetes (PD) was induced (see text for details) 4 weeks before intervention with a high-fat diet with (MPE) or without (PDC; disease control) mango “Ataulfo” peel extract and tracked for 6 and 8 weeks more. Fasting glucose (FG) and insulin (FI), total (TC), high (HDL-c), low (LDL-c), and very low (VLDL-c) lipoprotein cholesterol; Homeostatic model assessment of insulin resistance (HOMA-IR) and pancreatic β-cell reserve (HOMA-β), quantitative insulin sensitivity index (QUICKI).

## Data Availability

Not applicable.

## Supplementary Materials

The following supporting information can be downloaded at: https://www.mdpi.com/article/10.3390/life12040532/s1, Figure S1: Representative UPLC-DAD chromatogram used to identify and quantify phenolic compounds present in mango peel ethanolic extract; Figure S2: Food ingestion of murine model of prediabetes, Table S1: UPLC-DAD phenolic profile of mango peel extract (MPE).
