# Supplementary material for: Mango “Ataulfo” Peel Extract Improves Metabolic Dysregulation in Prediabetic Wistar Rats
Source: Life (Basel). 2022 Apr 5;12(4):532. doi: 10.3390/life12040532 (PMC9027361; doi:10.3390/life12040532)
Supplement: Supplementary file 1 [file life-12-00532-s001.zip › life-1660940-supplementary.pdf]

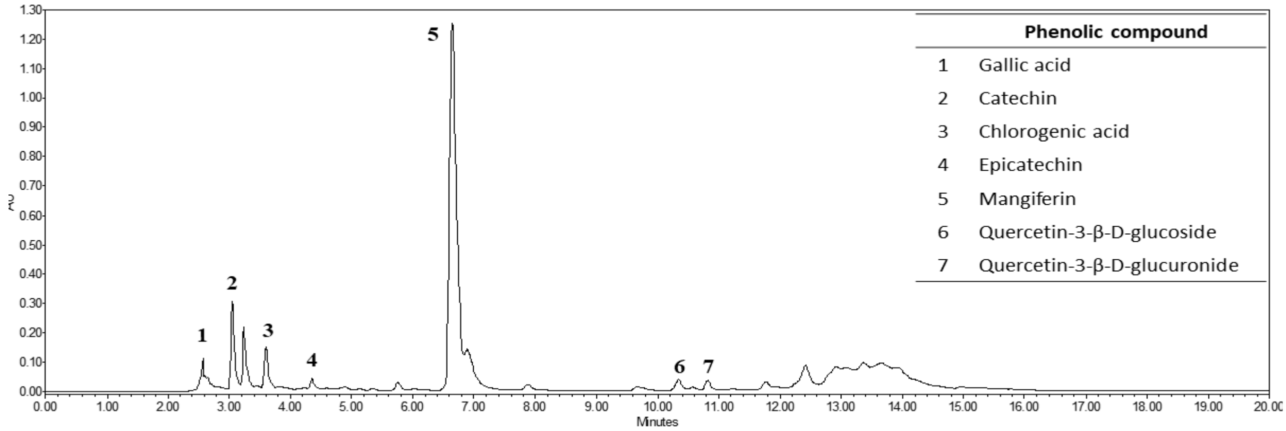

Figure S1. Representative UPLC-DAD chromatogram used to identify and quantify phenolic compounds present in mango peel ethanolic extract.

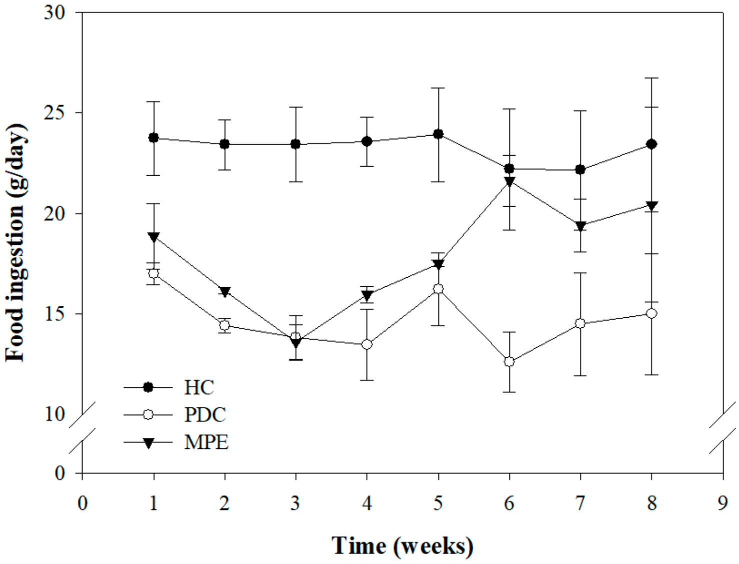

Figure S2. Time-trend changes in food ingestion of experimental groups. HC: healthy control; PDC prediabetes control group; MPE: mango peel ethanolic extract group.

Table S1. UPLC-DAD phenolic profile of mango peel extract (MPE).

Phenolic compound	Concentration (μg.g^{-1})
Gallic acid	1.57 ± 0.12
Catechin	0.63 ± 0.03
Chlorogenic acid	1.00 ± 0.04
Epicatechin	0.38 ± 0.07
Mangiferin	66.93 ± 5.64
Quercetin-3-β-D-glucoside	1.17 ± 0.06
Quercetin-3-β-D-glucoronide	0.04 ± 0.01

Results are expressed as mean ± standard deviation (n = 3).
